# Letter of Welcome

**DOI:** 10.1186/1617-9625-2-4-167

**Published:** 2004-12-15

**Authors:** Denis F Kinane

**Affiliations:** 1 Associate Dean for Research, University of Louisville, School of Dentistry

## 

October 26, 2004

Dear Conference Delegates,

It is with great pleasure that I offer a warm Louisville welcome to all delegates of the 2004 Annual Scientific Meeting of the *International Society for the Prevention of Tobacco Induced Diseases*.

Smoking kills 5 million people each year, an annual death toll that is expected soon to reach 10 million. Therefore, it is difficult to overestimate the importance of this global health concern. It is my sincere hope that the combined body of expertise assembling for this conference will contribute to reducing the tobacco-induced disease burden by disseminating the latest research findings, forming research collaborations, and assembling multidisciplinary alliances.

As you are aware Kentucky represents the heart of tobacco country and we have many lessons still to learn in the area of tobacco control. Thus, it is anticipated that the Louisville conference may help to promote tobacco control in our own region and, thus, have a long-lasting beneficial effect on our population.

During your visit, I do hope that you will find the time to relax and enjoy the wide range of activities that Louisville, and the surrounding countryside, has to offer.

Thank you for attending this important scientific conference, and again, welcome to Louisville.

Denis F. Kinane, BDS, PhD, FDS RCPS, FDS RCS

Delta Dental Endowed Professor

Associate Dean for Research and Enterprise

Continuing Health Sciences Education

To the "Third Annual Conference of the International Society of Tobacco Induced Diseases" (ISPTID) HOW TOBACCO KILLS

**Accreditation Statement**

The University of Louisville School of Medicine is accredited by the Accreditation Council for Continuing Medical Education to provide continuing medical education for physicians.

**Category 1 Credit**

Physicians wishing to receive Category 1 credit must complete and turn in the evaluation and registration for credit forms at the conclusion of the program. Please make sure your name, address, the last 4 digits of your Social Security number and license# are clearly printed on the form.

The University of Louisville Continuing Health Sciences Education designates this educational activity for up to 14.3 hours in Category 1 credit towards the AMA physicians Recognition Award. Each physician should claim only those hours of credit that he/she actually spent in the educational activity.

**Dental Credit**

Approved by the Kentucky Board of Dentistry for CE Credit as follows:

Saturday, October 30 – 7 Hours, Category B

Sunday, October 31 – 4.5 Hours, Category B

Monday, Nov. 1 – 3.0 Hours, Category B

**Nursing Credit**

This program has been approved by the Kentucky Board of Nursing for a total of 17.2 contact hours through the University of Louisville School of Nursing: Saturday, 9.4 hrs (Provider Number 3-0046-7-05-062), Sunday, 4.2 hours (Provider Number 3-0046-7-05-064) Monday, 3.6 hours (Provider Number 3-0046-7-05-065) expiration date July 1, 2005. The Kentucky Board of Nursing approval of an individual nursing education provider does not constitute endorsement of program content. Participants must attend entire session, provide license and social security number, and complete evaluation to receive contact hours.

**AAFP Credit**

This activity has been reviewed and is acceptable for up to **13.50 **Prescribed credit hours by the American Academy of Family Physicians.

**Disclosure**

As a sponsor accredited by the ACCME, the Office of Continuing Health Sciences Education, School of Medicine, University of Louisville, must insure balance, independence, objectivity, and scientific rigor in all its sponsored educational activities. All faculty participating in this CME Symposium were asked to disclose. **1**. No disclosure need be made for me since my presentation will not refer to products, devices or services, trade name or generic, of a commercial company with which I have a significant relationship **2**. for the following commercial companies whose products, devices or services I will refer to in my presentation I have a significant relationship **3**. If they have a significant relationship with the supporter of this activity; **4**. if they have a relationship with a commercial company not supporting this activity that might influence the information they provide in their presentation; **5**. if they will be discussing any product(s) that is still investigational or not labeled for the use under discussion and, if so, will they disclose this information to the activity audience. **6**. if they will be discussing any product that is not approved in the Untied States for the use under discussion; **7**. are the recommendations involving clinical medicine based on evidence that is accepted within the profession of medicine as adequate justification for their indications and contraindications in the care of patients; **8**. does all scientific research referred to, reported or used in support or justification of a patient care recommendation conform to the generally accepted standards of experimental design, data collection and analysis; **9**. they will use only generic names when discussing therapeutic options, if no, they will use trade names of several companies. Please note the following disclosures.

Ozan Akca, M.D. No relevant financial relationship(s) exist

*John Banzhaf, M.D. *No relevant financial relationship(s) exist

Parimal Chowdhury, Ph.D. No relevant financial relationship(s) exist

Jeffrey Ebersole, Ph.D. No relevant financial relationship(s) exist

Robert Genco, DDS, Ph.D. No relevant financial relationship(s) exist

David Hein, Ph.D. 3. Consultant-Cooperative Research Agreement/Procter and Gamble

Anthony Hedley, M.D. No relevant financial relationship(s) exist

Richard Hurt, M.D. **2**. Consultant/Sanofi-Synthelabo Research, Inc. (consultant fees for Dr. Hurt's time have been arranged through and paid to Mayo Foundation)

**5**. Yes

Dennis Kinane, BDS, Ph.D No relevant financial relationship(s) exist

Ernest Lam, DMD, Ph.D. No relevant financial relationship(s) exist

T.H. Lam, M.D. No relevant financial relationship(s) exist

Philip Lazarus, Ph.D. No relevant financial relationship(s) exist

Sarah McGhee, Ph.D. No relevant financial relationship(s) exist

Donald Miller, M.D. Disclosure not submitted before printing

Dennis Molfese, Disclosure not submitted before printing

Larry Palmer, LL.B. No relevant financial relationship(s) exist

Russell Prough, Ph.D. No relevant financial relationship(s) exist

Christine Ritchie, M.D. No relevant financial relationship(s) exist

Kazunari Satomura, M.D. No relevant financial relationship(s) exist

David Scott, Ph.D. No relevant financial relationship(s) exist

Jamie Studts, Ph.D. No relevant financial relationship(s) exist

David Tollerud, M.D. Disclosure not submitted before printing

Karen Waters, MBBS, Ph.D. No relevant financial relationship(s) exist

Jeffrey Wigand, Ph.D. Disclosure not submitted before printing

The sponsor acknowledges the following with special thanks for their support of this educational activity.

Procter and Gamble

## 

Please see additional files 1, 2, 3 and 4

## Competing interests

The authors declare that they have no competing interests.

## Supplementary Material

Additional file 1 Click here for file

Additional file 2 Click here for file

Additional file 3 Click here for file

Additional file 4 Click here for file

