# Supplementary material for: Letter of Welcome
Source: Tob Induc Dis. 2004 Dec 15;2(4):167. doi: 10.1186/1617-9625-2-4-167 (PMC2691732; doi:10.1186/1617-9625-2-4-167)
Supplement: Additional file 1 [file 1617-9625-2-4-167-S1.pdf]

This Conference is generously supported by the following institutes and organizations:

University of Louisville School of Dentistry

Procter & Gamble
