# Supplementary material for: Letter of Welcome
Source: Tob Induc Dis. 2004 Dec 15;2(4):167. doi: 10.1186/1617-9625-2-4-167 (PMC2691732; doi:10.1186/1617-9625-2-4-167)
Supplement: Additional file 3 [file 1617-9625-2-4-167-S3.pdf]

Conference Overview

Friday, October 29th

Conference Reception at University of Louisville School of Dentistry 19.00 - 22.00
(501 S. Preston St.)
Posters may be mounted for display at this time and remain in place for the Sunday evening poster session.

Saturday, October 30th

Seelbach Hilton Hotel (Page)

REGISTRATION: The onsite registration desk will open at 08.00
(Continental Breakfast available)

Session I: Public Health Issues 08.30 – 12.00 (9)

Session II: General Pathogenic Mechanisms 13.30 – 16.00 (14)

Session III: Specific Disease-Related Mechanisms 16.20 – 17.30 (19)

CONFERENCE DINNER in the Seelbach Hilton Ballroom 19:30
(Prepaid Registration Required; Additional dinner tickets available until NOON only)

Sunday, October 31st

REGISTRATION: The onsite registration desk will open at 08.00
(Continental Breakfast available)

Session III: Specific Disease-Related Mechanisms (cont.) 08.30 – 12.30 (22)

Afternoon Recreation at Churchill Downs

Poster Session at University of Louisville School of Dentistry 18.00 – 21.00 (35)

Monday, November 1st

Session IV: Tobacco Cessation and Policies 08.30 – 12.00 (29)

ISPTID BUSINESS SESSION 13.00 – 15.00
(All Members are invited to attend.)
