# Supplementary material for: Letter of Welcome
Source: Tob Induc Dis. 2004 Dec 15;2(4):167. doi: 10.1186/1617-9625-2-4-167 (PMC2691732; doi:10.1186/1617-9625-2-4-167)
Supplement: Additional file 4 [file 1617-9625-2-4-167-S4.pdf]

Poster Session:

University of Louisville School of Dentistry
501 S. Preston Street

Sunday, October 31st
6.00 pm - 9.00 pm

Poster Judges:

Dr. T.H. Lam, University of Hong Kong
Dr. David W. Hein, University of Louisville, USA
Dr. Kazunari Satomura, Kyoto University, Japan

POSTERS:

21. Predictors of intention to quit and successful quitting among the Chinese elderly smokers

Abu Saleh M. Abdullah*[1], Lai-Ming Ho[1], Yam H Kwan[1], Cheung WL[2], McGhee SM[1].
[1]Department of Community Medicine, The University of Hong Kong, Hong Kong SAR, China.
[2]Caritas Medical Centre, Hong Kong SAR, China.

22. Predictors of household smoking policies among San Diego residents of Mexican descent

Marc A. Adams MPH, Ana P. Martinez-Donate PhD, Melbourne F. Hovell PhD, C. Richard Hofstetter PhD, Carlos Vera, Gloria Valerio
Center for Behavioral Epidemiology and Community Health Graduate School of Public Health, San Diego State University.

23. The effects of cigarette smoke on hepatic and pulmonary xenobiotic metabolizing enzymes in rats

B.C. Eke , M. __can*
Department of Toxicology, Faculty of Pharmacy, Ankara University, 06100 Tando_an- Ankara, TURKEY

24. Epidemiology and prevention of tobacco use in Tunisia

Radhouane FAKHFAKH*, Mohamed HSAIRI, Noureddine ACHOUR
Institut Naitonal de Sante Publique, 5-7 rue Khartoum IMM Diplomat 1002 Le belvedere, Tunis

25. Two week nicotine treatment selectively increases bone vascular constriction in response to norepinephrine

Fleming J.T., J.B.A. Feitelson, P.P. Rowell, C.S. Roberts
Departments of Physiology and Biophysics and Pharmacology and Toxicology, University of Louisville, Louisville, Kentucky, USA

26. Evaluation of a statewide population-based cessation contest

Ellen J. Hahn, DNS, RN; Mary Kay Rayens, PhD; Dawn M. Christie, MA; Lisa W. Greathouse, MSN, RN; Nancy L. York, MSN, RN
College of Nursing • University of Kentucky • Lexington, Kentucky

27. Reducing secondhand tobacco smoke: Cardiac and asthma outcomes

Ellen J. Hahn, DNS, RN; Debra K. Moser, DNSc, RN, FAAN; Patricia Burkhart, PhD, RN; Mary Kay Rayens, PhD; Nancy L. York, MSN, RN
College of Nursing • University of Kentucky • Lexington, Kentucky

28. Underestimation of mortality due to chronic obstructive pulmonary disease (COPD) in Kentucky

Therese S. Hughes, MPA, Susan B Muldoon, PhD, MPH
University of Louisville School of Public Health and Information Sciences
Department of Epidemiology and Clinical Investigation Sciences

29. Tobacco compromises periodontal health in a maintenance population

Kells L*[1], Picard J-P[1], Gelskey SC[1], Lix L[2], Singer DL[1], Scott DA[1,3]
Depts. Of DDSS[1], Community Health Medicine[2], and Oral Biology[3], University of Manitoba, Canada

30. Friends, family, and the school environment: a multi-level analysis of their relationship to student smoking behaviour

Scott Leatherdale, PhD
Cancer Care Ontario, Toronto, Ontario, Canada

31. Knowledge, attitudes, and clinical practices involving tobacco cessation among Kentucky physicians

J.L. Marmorato*[1], C.T. Worth[2], J.L. Studts[1], and C.L. Sorrell[2]
[1]University of Louisville School of Medicine, [2]Kentucky Cancer Program

32. Smoking, exposure to environmental tobacco smoke (ETS), and home policies on smoking in the border city of Tijuana, Mexico

Ana P. Martinez-Donate*, Melbourne F. Hovell, Marc A. Adams, Jose de Jesus Sanchez, Margarita Viñas, Gabriela Guzman, Jesus R. Gonzalez, Ernesto Moreno Almaraz, M. Guadalupe Sanchez Hernandez, Oscar I. Jimenez Garcia
Center for Behavioral Epidemiology and Community Health Graduate School of Public Health, San Diego State University.

33. Development of a Computer Program for Translation of human N-acetyltransferase-1 and -2 SNP data into genotype and phenotype: Applications to tobacco use risk assessment

Benjamin D. Martini*, Mark A. Doll and David W. Hein
Department of Pharmacology & Toxicology and James Graham Brown Cancer Center, University of Louisville School of Medicine, Louisville, Kentucky USA

34. Tobacco smoke components and metabolites alter fetal and neonatal lung cell function

J. Merluza*, C. Hillier, B. Weltman, P. Irani, X. Xie, D.A. Scott, and J.E. Scott
Department of Oral Biology, Faculty of Dentistry, University of Manitoba & Biology of Breathing Group, Manitoba Institute of Child Health, Children's Hospital Research Foundation, Winnipeg, Canada

35. Knowledge, attitudes, and clinical practices involving tobacco cessation among Kentucky psychologists

B.F. Miller*[1], C.S. Lotz[1], C.T. Worth2, C.L. Sorrell[2], and J.L. Studts[3]
[1]Spalding University, [2]Kentucky Cancer Program, [3]University of Louisville School of Medicine

36. Cigarette smoke condensate in oral cancer – apoptosis or inflammation?

Nagathihalli S. Nagaraj1, Nadaraj Vigneswaran[3], and Wolfgang Zacharias[1,2]
[1]Department of Medicine, James Graham Brown Cancer Center, [2]Department of Pharmacology & Toxicology, University of Louisville, Louisville, Kentucky 40202, USA, [3]Departments of Diagnostic Sciences, The University of Texas Health Science Center at Houston, Dental Branch, Houston, Texas 77030, USA

37. Acute nicotine treatment does not reduce blood flow to rat mandibles

*Pandya, P., J.T. Fleming, and P.P. Rowell
Departments of Physiology and Biophysics and Pharmacology and Toxicology, University of Louisville, Louisville, KY, USA

38. Effects of tetracyclines on biomarkers of systemic inflammation in smokers with periodontitis

Simon, S.*, Roemer, E., Camu, O., Farrell, J., Tenzler, R and Ryan, M.E.
Stony Brook University, Stony Brook, New York

39. Association of smoking with increasing vascular involvement in Type 2 diabetic Chinese patients

GN Thomas[1*]; B Tomlinson[2]; Sarah M McGhee[1]; ASM Abdullah[1]; V Yeung[2], JCN Chan[2] and KS Wong[2].
Department of Community Medicine[1], University of Hong Kong, Pokfulam, Hong Kong SAR
Department of Medicine and Therapeutics[2], The Chinese University of Hong Kong, The Prince of Wales Hospital, Shatin, Hong Kong

40. Knowledge, attitudes, and clinical practices involving tobacco cessation among Kentucky dentists

*S.D. Thomas[1], C.T. Worth[2], J.L. Studts[1], and C.L. Sorrell[2]
[1]University of Louisville School of Medicine, [2]Kentucky Cancer Program

41. Predictors of smoking initiation and smoking patterns among college students: Personality factors, sense of coherence, self-efficacy, & social support

Diane Von Ah, PhD, RN, University of Louisville, School of Nursing & Sheryl Ebert, PhD Candidate, University of Alabama at Birmingham, Department of Psychology

42. Fetal mandibular condyle cell Matrix Metalloproteases (MMPs) secretions are altered by tobacco smoke components and metabolites

B. Weltman,* J. Merluzza, C. Hillier, P. Irani, X. Xie, D.A. Scott, and J.E. Scott
Department of Oral Biology, Faculty of Dentistry, University of Manitoba & Manitoba Institute of Child Health, Children's Hospital Research Foundation, Winnipeg, Canada

43. Treating tobacco use and dependence: One year follow-up of the Providers Practice Prevention Program

* Celeste T. Worth[1], Jamie L. Studts[2], and Connie L. Sorrell[1]
[1]Kentucky Cancer Program-University of Louisville, [2]University of Louisville School of Medicine

44. How do daily and occasional smokers differ in quit behaviour?

*Anne Marie Zaborski, B.A., Senior Survey Methodologist, Office of Research, Surveillance and Evaluation, Tobacco Control Programme, Health Canada
Judy Snider, M.Sc., Manager of Surveillance, Office of Research, Surveillance and Evaluation, Tobacco Control Programme, Health Canada
Caren Uhlik, B.P.E., Analyst, Office of Research, Surveillance and Evaluation, Tobacco Control Programme, Health Canada

45. The influence of nicotine on microlymphatics in rat mesentery

V.P. Zharov[a], E.I. Galanzha [a,b], *P. Chowdhury [c], V.V. Tuchin [b]
[a]Philips Classic Laser Laboratories, University of Arkansas for Medical Sciences (UAMS), Little Rock, AR, 72205
[b]Saratov State University, Saratov, Russia
[c]Department of Physiology and Biophysics, UAMS, Little Rock, AR, 72205
